# Co-morbidities are the key nominators of the health related quality of life in mild and moderate COPD

**DOI:** 10.1186/1471-2466-14-102

**Published:** 2014-06-19

**Authors:** Jukka Koskela, Maritta Kilpeläinen, Henna Kupiainen, Witold Mazur, Harri Sintonen, Marike Boezen, Ari Lindqvist, Dirkje Postma, Tarja Laitinen

**Affiliations:** 1 Clinical Research Unit for Pulmonary Diseases and Division of Pulmonology, Helsinki University Central Hospital, Helsinki, Finland; 2 Division of Medicine, Department of Pulmonary Diseases and Clinical Allergology, Turku University Hospital and University of Turku, Turku, Finland; 3 Department of Public Health, University of Helsinki, Helsinki, Finland; 4 University of Groningen, University Medical Center Groningen, GRIAC Research Institute, Groningen, The Netherlands; 5 Research Unit for Pulmonary Diseases, Helsinki University Central Hospital, Tukholmankatu 8C, PO Box 705, FI-00029 HUS Helsinki, Finland

**Keywords:** Generic HRQoL, Respiratory specific HRQoL, HRQoL, COPD, 15D, AQ20, Co-morbidities, Mortality

## Abstract

**Background:**

Co-morbidities are common in chronic obstructive pulmonary disease (COPD). We assessed the contribution of common co-morbidities on health related quality of life (HRQoL) among COPD patients.

**Methods:**

Using both generic (15D) and respiratory-specific (AQ20) instruments, HRQoL was assessed in a hospital based COPD population (N = 739, 64% males, mean age 64 years, SD 7 years) in this observational study with inferential analysis. The prevalence of their co-morbidities was compared with those of 5000 population controls. The patients represented all severity stages of COPD and the patterns of common concomitant disorders differed between patients.

**Results:**

Co-morbidities such as psychiatric conditions, alcohol abuse, cardiovascular diseases, and diabetes were more common among COPD patients than in age and gender matched controls. Psychiatric conditions and alcohol abuse were the strongest determinants of HRQoL in COPD and could be detected by both 15D (Odds Ratio 4.7 and 2.3 respectively) and AQ20 (OR 2.0 and 3.0) instruments. Compared to respiratory specific AQ20, generic 15D was more sensitive to the effects of comorbidities while AQ20 was slightly more sensitive for the low FEV_1_. FEV_1_ was a strong determinant of HRQoL only at more severe stages of disease (FEV_1_ < 40% of predicted). Poor HRQoL also predicted death during the next five years.

**Conclusions:**

The results suggest that co-morbidities may impair HRQoL at an early stage of the disease, while bronchial obstruction becomes a significant determinant of HRQoL only in severe COPD.

## Background

Chronic obstructive pulmonary disease (COPD) is globally one of the leading causes of morbidity, disability, and mortality according to World Health Organization (http://www.who.int/respiratory/copd/en/). For decades, COPD was considered simply as a disease with progressive airflow limitation. Lately it has become obvious that COPD patients suffer simultaneously from several extra-pulmonary diseases such as cardiovascular diseases, diabetes, osteoporosis, musculoskeletal dysfunction, chronic kidney and heart failure, anxiety, depression, and addiction diseases [1-5]. In several population studies both increased morbidity and mortality are associated with lung function impairment [6-13]. Furthermore, co-morbidities have additional impact on health, for example the response to treatment received for coronary artery disease is worse in patients with co-existing COPD [14,15]. The underlying causes of multi-morbidity in COPD are still poorly understood. The co-occurrence of the diseases may appear simply due to patients’ aging, or due to shared risk factors or etiological pathways. The latter is compatible with the hypothesis that COPD sustains systemic inflammation in the body [4].

Spirometry alone is not enough in clinical practice to predict the future outcome of COPD as for instance exacerbation rates and co-morbidity may contribute as well [16]. This has initiated studies to develop a multidimensional score with a better predictive power to monitor progression of COPD than FEV_1_ alone. Health related quality of life (HRQoL) has become one of the components of interest. So far our experience on HRQoL in COPD has mainly emerged from randomised clinical trials in which HRQoL is frequently evaluated as a secondary end point [17,18]. Contrary to clinical practice, the severity of COPD is often restricted and patients with severe co-occurring diseases are under-represented or even excluded. Traditionally, the number of female patients has been limited in clinical trials as well, while it is known that this affects the prevalence of co-morbidities [19] and treatment decisions [20]. Therefore, determining the effect of co-morbidities and gender on HRQoL has become increasingly important also in real world observational studies. There is also a need for the development of programs that enhance supportive care for patients with severe COPD [21]. This is particularly important since HRQoL may offer a good outcome-measure for holistic interventional studies that take into account patient’s physical, psychological, social and spiritual well-being.

In the present study we aim to estimate the contribution of common co-morbidities on self-reported HRQoL among COPD patients by using two questionnaires: the AQ20 focusing on respiratory health [22] and the 15D focusing on generic health [23]. Both questionnaires are simple and short, and can be used easily in clinical practice with high response and completion rates [24,25]. AQ20 has discriminative properties and responsiveness comparable to more complex questionnaires such as SGRQ [24], and 15D and AQ20 have convergent validity to measure HRQoL in COPD [25]. We also studied the association between HRQoL and the 5-year-mortality.

## Methods

### Subjects

Hospital Discharge Registries were used to identify all patients with COPD who had visited the Pulmonary Clinics of the Helsinki and Turku University Hospitals during the years 1995–2006. The databases were screened by ICD10 code J44.8 and contained all patients between 18 to 75 years of age. The upper level was selected due to our study design, which included a 10-year follow-up. All identified patients were invited to the study without further selection. The recruitment was done through a two-phase mailing campaign, through which altogether 844 patients participated the study [26], after which their diagnosis was carefully assessed going through the medical records by a pulmonologist. This resulted in 739 confirmed COPD patients (clinical symptoms and airway obstruction according GOLD) with smoking related disease. The enrolment occurred during the years 2005–2007. All the participants gave informed consent to collect, merge, and analyse their comprehensive medical history from all the healthcare providers who had treated them during the past 5–10 years. Participants of the Health2000 survey (N = 5000) were used as age and gender matched population controls to avoid biased inferences. Their diagnostic data had been gathered from the hospital discharge registries (http://www.terveys2000.fi/indexe.html).

### Assessment of HRQoL

The HRQoL was assessed using the self-administered Airways Questionnaire 20 [27] and 15D (http://www.15d-instrument.net/15D). All participants filled in both questionnaires during the research visit without any guidance from the research personnel. The AQ20 questionnaire was used as the disease-specific instrument of HRQoL. This questionnaire was developed in 1998 for use in asthma [27] and COPD [22,28]. The AQ20 is a uni-dimensional measure containing 20 items with “yes” responses are scored as 1, and “no” and “not applicable” are scored as 0. The scores of 1 are summed up to obtain the AQ20 summary score, which ranges from 0 to 20, a score of zero indicating no respiratory symptoms or worries about the disease. The 15D–instrument [29] is a generic, multidimensional, standardized, self-administered evaluative tool of HRQoL that can be used primarily as a single index measure, but also as a profile measure. It describes the health status, with 15 dimensions, namely: mobility, vision, hearing, breathing, sleeping, eating, speech, excretion, usual activities, mental function, discomfort and symptoms, depression, distress, vitality, and sexual activity. Each dimension comprises one question on a 5-point scale. A single index score is obtained by incorporating population-based preference weights to the 15 dimensions. The maximum score is 1 (no problems on any dimension) and the minimum score is 0 (being dead). The 15D instrument has good test–retest reliability, construct validity, and discriminatory power in general populations [23] and has been validated for use in COPD [30,31]. All participants returned the questionnaire at the recruitment session. There was no missing data for AQ20, for 15D eight patients did not finish the questionnaire completely and this data was omitted from analysis. Recall bias was not assessed in this study.

### Physiological measurements and co-morbid conditions

All hospitals, health care centres, and other outpatient clinics that had treated the patients were contacted to archive a complete medical history for each participant. The patients’ social security number was used to combine the data from different sources. Source data with personal identifiers were managed and stored in the Clinical Research Centres administered by the Helsinki and Turku University Central Hospitals. The results of flow-volume spirometry including bronchodilatation tests nearest to index date, diffusion capacity, ECGs at rest, weight, height and smoking status were used in the analysis. The reference values used for FEV_1_ and FVC in Finnish clinical practice were validated in large Finnish population samples consisting of both genders and a wide range of age groups [32]. All the given diagnoses stated in the medical records were carefully evaluated; especially concerning the time of onset and certainty of the diagnosis was determined. Category ‘coronary disease’ included the patients who have had a myocardial infarction, acute coronary artery syndrome, or angina pectoris diagnosed by an internist or cardiologist. ‘Cerebrovascular diseases’ included patients with strokes and transient ischemic attacks diagnosed by a neurologist. ‘Cardiovascular diseases’ consisted of patients having one of the following diseases: coronary, cerebrovascular disease, or peripheral artery occlusive disease. Chronic atrial fibrillation was confirmed by ECG recordings. Category ‘diabetes’ includes patients with the type 1 and 2 disease. Chronic alcoholism, alcohol use disorder, and treatment of alcohol-use related disorders were categorized as ‘alcohol abuse’. A wide range of psychotic disorders and long lasting clinical depression, and anxiety that needed regular medication were categorized as ‘Psychiatric condition’. Category ‘cancer’ included all malignant solid tumours and malignant haematological diseases. The prevalence of the common co-existing diseases were compared with an age and gender matched control population from a Finnish population survey (Health 2000, N = 5000) [33]. Diffusing capacity and ECG were available from a limited number of the patients and therefore excluded from the multivariate regression analyses (Table 1 and Additional file 1: Table S3). The modified Medical Research Council (mMRC) dyspnoea scale was used to measure subjective breathlessness [34].

**Table 1:** Risk factors for low HRQoL (AQ20 summary score ≥14) based on univariate and backwards-stepwise multivariate regression analysis

**Patient characteristics**	**N of patients analyzed**	**% patients with poor HRQoL**	**Crude OR**	**95%** ***CI***	**P-value**	**Adjusted OR**	**95%** ***CI***	**P-value**
Gender								
Male	422	10.8	1.00					
Female	225	15.4	1.51	0.97-2.35	NS	**2.11**	**1.27-3.51**	**0.004**
Age at recruitment								
<60 years	215	13.5	1.00					
61–68 years	274	14.2	1.06	0.64-1.79	NS			
>69 years	250	9.6	0.68	0.38-1.21	NS			
FEV_1_ baseline % of predicted								
>80	*95*	9.5	1.00					
65–80	*185*	4.9	0.50	0.17-1.43	NS	0.54	0.18-1.63	0.27
40–64	*323*	13.3	1.47	0.63-3.43	NS	**2.48**	**0.99-5.95**	**0.05**
<40	*136*	22.9	**2.84**	**1.19-6.79**	**0.02**	**5.17**	**2.03-13.17**	**0.001**
Smoking cessation succeed								
Yes	358	11.2	1.00					
No	281	14.2	1.32	0.83-2.11	NS			
BMI units								
<19	33	34.3	-	-	-			
19–25	251	4.5	1.00					
>25-30	275	37.6	0.64	0.37-1.07	NS			
>30	173	23.6	0.95	0.54-1.64	NS			
Diabetes								
No	628	11.9	1.00					
Yes	111	15.3	1.33	0.75-2.36	NS			
Cardiovascular Disease^1^								
No	532	13.3	1.00					
Yes	207	10.1	0.73	0.44-1.23	NS			
Hypertension								
No	439	12.3	1.00					
Yes	300	12.7	1.03	0.66-1.61	NS			
Atrial Fibrillation						-^2^		
No	474	14.1	1.00					
Yes	31	29.0	**2.49**	**1.10-5.63**	**0.03**			
Diffusing capacity								
Normal	221	11.8	1.00					
Mild/moderately decrease	89	11.2	0.95	0.44-2.06	NS			
Severe decrease	103	16.5	1.48	0.77-2.87	NS			
Cancer								
No	695	12.4	1.00					
Yes	44	13.6	1.12	0.46-2.72	NS			
Psychiatric conditions								
No	493	9.9	1.00					
Yes	240	17.1	**1.87**	**1.19-2.92**	**0.006**	**1.99**	**1.21-3.29**	**0.007**
Alcohol abuse								
No	629	10.5	1.00					
Yes	110	23.6	**2.64**	**1.59-4.39**	**<0.001**	**3.02**	**1.65-5.51**	**0.001**

Odd Ratios with P values ≤0.05 are shown in bold.

^1^coronary, cerebrovascular, or peripheral artery disease, ^2^due to small number of tested patients not included to the multivariate analysis.

The Coordinating Ethics Committee of the Helsinki and Uusimaa Hospital District approved the study approach, and the permission to conduct this research was granted by the Helsinki and Turku University Hospitals. All the clinical studies, sample collection and storage, and data gathering processes were done according to the standardised operational procedures (SOPs), the training of the study personnel has been documented and done according to good clinical practice. Clinical study processes have been monitored after enrolment and after each annual follow-up contact with the study subject.

### Statistical analysis

Analyses were performed by the statistical software packages SPSS (IBM Corp. Released 2011. IBM SPSS Statistics for Windows, Version 20.0. Armonk, NY: IBM Corp) and R Statistical Software (R Development Core Team (2008), R version 2.15, http://www.R-project.org).

Correlations of 15D and AQ20 scores with lung function measurements were analysed using the Spearman correlation coefficient. Both HRQoL scores were analysed as continuous and as categorised variables. Analysis of continuous endpoints enables inferences throughout the scale of HRQoL, while categorised endpoints only compare differences between two groups. Thus parallel analysis of categorised and continuous endpoints was used as supportive approach to confirm congruent results. Linear regression was used to determine clinically valid attributes potentially affecting HRQoL and to compare their relative importance. Regression coefficient B is the change in HRQoL when dependent variable changes with one unit. To quantify the effect of a particular dependent variable we determined standardized regression coefficient β, which is a modification of B. The effect is standardized from -1 to 1 allowing solid comparison between the variables. Differences in means of FEV_1_ % of predicted were determined using ANOVA combined with Tukey's Honestly Significant Difference.

The 10.0% (in 15D) and 12.4% (closest discrete value to 10% in AQ20) of patients having the lowest HRQoL score formed the low-HRQoL subgroup. Logistic backwards-stepwise regression was used to determine the independent risk factors for low HRQoL. All covariates presented as crude ORs (Tables 1 and 2) were included into backwards-stepwise logistic regression to construct a multivariate model defining independent risk factors for poor HRQoL. The model constructed for poor 15D was then used to assess its performance in predicting 5-year mortality by determining Receiver Operating Characteristic (ROC) and Area Under the Curve (AUC). The Minimal Clinically Important Difference (MCID) in 15D total score is 0.03 [35]. Analyses were done separately for both 15D (Table 2) and AQ20 (Table 1).

**Table 2:** Risk factors for a low score on 15D (total score ≤0.65) based on univariate and backwards stepwise multivariate regression analysis

**Patient characteristics**	**N of patients analyzed**	**% patients with poor HRQoL**	**Crude OR**	**95%** ***CI***	**P-value**	**Adjusted OR**	**95%** ***CI***	**P-value**
Gender								
Male	470	9.6	1.00					
Female	261	11.1	1.18	0.72-1.93	NS			
Age at recruitment								
<60 years	214	9.8	1.00					
61–68 years	269	10.4	1.97	0.59-1.94	NS			
>69 years	248	10.1	1.03	0.56-1.90	NS			
FEV_1_ baseline % of predicted								
>80	*94*	8.2	1.00			1.00		
65–80	*184*	7.5	0.91	0.33-1.39	NS	1.02	0.35-3.01	NS
40–64	*318*	9.8	1.21	0.53-1.88	NS	1.41	0.52-3.80	NS
<40	*135*	16.5	2.21	0.86-5.71	0.100	**3.08**	**1.10-8.63**	**0.03**
Smoking cessation succeed								
Yes	355	7.9	1.00					
No	278	12.6	**1.68**	**1.00-2.84**	**0.05**			
BMI units								
<19	32	9.3	0.65	0.15-2.91	NS			
19–25	248	6.2	1.00					
>25-30	272	9.2	0.99	0.55-1.79	NS			
>30	172	14.0	1.59	0.86-2.92	NS			
Diabetes								
No	620	8.4	1.00			1.00		
Yes	111	19.8	**2.70**	**1.56-4.66**	**<0.001**	**2.12**	**1.07-4.22**	**0.03**
Cardiovascular Disease^1^								
No	526	9.1	1.00			1.00		
Yes	205	12.7	1.45	0.87-2.40	NS	1.69	0.93-3.07	0.09
Hypertension								
No	434	9.4	1.00					
Yes	297	11.1	1.20	0.74-1.94	NS			
Atrial Fibrillation								
No	468	11.1	1.00			-^2^		
Yes	30	23.3	**2.44**	**1.00-5.95**	**0.05**			
Diffusing capacity								
Normal	220	10.0	1.00					
Mild/Moderately decrease	87	13.8	1.44	0.68-3.05	NS			
Severe decrease	102	11.8	1.20	0.57-2.53	NS			
Cancer								
No	687	10.0	1.00					
Yes	44	11.4	1.15	0.44-3.01	NS			
Psychiatric conditions								
No	488	5.9	1.00			1.00		
Yes	237	18.1	**3.51**	**2.13-5.78**	**<0.001**	**4.65**	**2.57-8.40**	**<0.001**
Alcohol abuse								
No	621	8.4	1.00			1.00		
Yes	110	20.0	**2.74**	**1.58-4.73**	**<0.001**	**2.33**	**1.21-4.49**	**0.007**

Odd Ratios with P values ≤0.05 are shown in bold.

^1^coronary, cerebrovascular, or peripheral artery disease, ^2^due to small number of tested patients not included to the multivariate analysis.

## Results

### Study subjects

A total of 739 eligible patients (64% male, mean age 64 ± SD 7 years, mean pack years 51 **±** SD 20) with COPD attended the study. The patients were identified through a two-phase mailing campaign, which was directed to all elderly asthma, and COPD patients who had visited the University Hospital of Helsinki and Turku years 1995–2006 [26]. The diagnosis was made on average 5.5 years before the enrolment. The severity of the disease was determined by the latest flow volume spirometry, which was performed within two years before the recruitment. According to the GOLD criteria [36], the majority of the patients belonged to the stages 2–3, moderate to severe COPD.

Even though women had slightly better lung function (mean FEV_1_ 55 vs. 61% of predicted, p < 0.001), they reported higher scores for several questions related to respiratory symptoms and the mean AQ20 summary score was significantly higher among women than men (7.9 vs. 8.8 p = 0.02) (Additional file 1: Table S2). For 15D the total score did not differ (0.79 for both sexes). Women, however, reported more difficulties with sleep, depression, and distress than men. Men suffered more frequently from impairment of sexual performance than women (Additional file 1: Table S1).

### Co-morbidities in the cohort

Common co-morbidities of the COPD patients were evaluated from the medical records (Table 3). Cardiovascular diseases, diabetes, and chronic psychiatric conditions were more prevalent in the COPD cohort than among the gender and age matched Finnish controls.

**Table 3:** Prevalence of co-morbidities in the COPD cohort compared to those in an age and gender matched Finnish population survey

**Chronic disease**	**COPD cohort N = 739**			**Finnish population cohorts health 2000N = 5000**	
	**Men %**	**Women %**	**Total %**	**Total %**	**Difference in prevalence**
Hypertension	41	40	41	-	
Coronary disease	27	12	22	16	1.4 x
Cerebrovascular disease	9	5	8	5^1^	1.6 x
Peripheral artery disease	6	2	5	2	2.5 x
Diabetes	18	11	15	9	1.7 x
Alcohol abuse	18	9	15	-	
Chronic psychiatric conditions	26	45	33	12	2.8 x
Cancer	6	6	6	7	0.9 x

^1^stroke including also intracerebral and subarachnoidal hemorrhage.

The prevalence of co-occurrence of the chronic medical conditions was comparable in males and females, no co-morbidities 27.9% vs. 26.3%, 1 co-morbidity 31.5 vs. 31.9%, 2 co-morbidities 21.4% vs. 26.3%, 3 co-morbidities 14.6% vs. 11.7% and ≥4 co-morbidities 4.7% vs. 3.8% respectively. The number of chronic conditions did not significantly associate with FEV_1_ levels.

### Lung function and co-morbidities in relation to HRQoL scores

Both FEV_1_ and diffusing capacity correlated with the HRQoL scores weakly or moderately, FEV_1_ slightly stronger than diffusing capacity and both lung function measurement slightly stronger to AQ20 than 15D (Table 4). The strongest correlation was found between the two HRQoL instruments (r = -0.69). The lung function measures (FEV_1_ and DLCOcVA) were also moderately correlated with each other (r = 0.35).

**Table 4:** Correlation matrix for lung function parameters, HRQoL, MRC Dyspnoea scale and age

	**15D score**	**Age**	**DLCOcVA**	**FEV1%**	**MRC scale**	FEV1*DLCOcVA	**AQ20 score**
**15D Score**	1	0.01	0.11*	0.16***	-0.03	0.15**	-0.69***
**Age**	0.01	1	-0.07	-0.19***	0.02	-0.17**	-0.09
**DLCOcVA**	0.11*	-0.07	1	0.35***	0.03	0.79***	-0.19***
**FEV1%**	0.16**	-0.19***	0.35***	1	0.00	0.83***	-0.24***
**MRC Scale**	-0.03	0.02	0.03	0.00	1	0.01	0.07
FEV1*DLCOcVA	0.15**	-0.17**	0.79***	0.83***	0.01	1	-0.25***
**AQ20 Score**	-0.69***	-0.09	-0.19***	-0.24***	0.07	-0.25***	1

*p-value < 0.05, **p < 0.01, ***p < 0.001.

Only a very small proportion of variance (adjusted R^2^ 3.5%) found in the 15D total score was explained by FEV_1_ and diffusion capacity alone. When co-morbidities were added into the model, psychiatric conditions, FEV_1_, alcohol abuse, diabetes, cardiovascular diseases, and hypertension were significant determinants of the total 15D scores. Psychiatric conditions (p ≤ 0.001) and FEV_1_ (p ≤ 0.001) had the greatest effect (β > 0.2). Alcohol abuse (p ≤ 0.001), cardiovascular diseases (p = 0.006), and diabetes (p = 0.007) had β-values in the range of 0.1 – 0.2 while hypertension (p = 0.04) had the smallest β (0.08). After performing stepwise regression, explained variance in 15D was increased considerably from 3.5% to 17.6%.

Correspondingly for AQ20, the greatest effect was found for FEV_1_ (β = 0.34, p < 0.001), while psychiatric conditions (p ≤ 0.001), female gender (p ≤ 0.001), alcohol abuse (p = 0.002) all had β-values in the range of 0.1 – 0.2, and hypertension had the smallest effect (β <0.1, p = 0.008). The model including the comorbidities explained 15.5% of the AQ20 summary score, while FEV_1_ and diffusion capacity alone explained only 6.9% of the variation.

### Clinical variables explaining low HRQoL

The mean 15D total score in the cohort was 0.79 (SD 0.11). Patients with the lowest 10% of the 15D score (N = 79/731, 9.6% of men and 11.1% of women) formed the low 15D category (total score ≤0.65, Table 2). Diabetes (OR 2.1, 95% CI 1.1-4.2, p = 0.03), psychiatric conditions and alcohol abuse (OR 2.3, 95% CI 1.2-4.5, p = 0.007), were independently associated with a low 15D score. The greatest risk of a low 15D score (OR 4.7, 95% CI 2.6-8.4, p ≤ 0.001) was shown among COPD patients with psychiatric conditions. A low 15D score was significantly related to airway obstruction only when the patients’ FEV_1_ was <40% of predicted. The OR of low 15D score was then three-fold compared to patients with FEV_1_ > 80% (95% CI 1.1-8.6, p = 0.03).

The mean AQ20 summary score in the cohort was 8.25 (SD = 5.03). The lowest 12.4% of AQ20 (summary score >14, N = 92/739, 12.1% of men and 18.2% of women) was associated with borderline significance to moderate airway obstruction (OR 2.5, 95% CI 1.0-6.0, p = 0.05, when FEV_1_ 64-40% of predicted) and significantly to severe airway obstruction (OR 5.2, 95% CI 2.0-13.2, p ≤ 0.001, when FEV_1_ < 40%). Alcohol abuse (OR 3.0, 95% CI 1.7-5.5, p ≤ 0.001) and psychiatric conditions (OR 2.0, 95% CI 1.2-3.3, p = 0.007) were the significant co-morbidities associated with poor AQ20 alongside with female gender (OR 2.1, 95% CI 1.3-3.5, p = 0.004). Contrary to 15D, cardiovascular diseases and diabetes remained non-significant predictors for poor AQ20 score (Table 1). Thirty-three patients were categorised having low HRQoL by both questionnaires.

The mMRC dyspnoea scale did not remain significant in any of the models after stepwise model selection.

### Mortality and HRQoL

In order to study the importance of the identified risk factors of low generic HRQoL, the same variables were used in a 5-year mortality model (N_total_ = 670, N_dead_ = 105) (Additional file 1: Table S3). FEV_1_ < 40% of predicted was the most important risk factor (OR 6.91, 95% CI 2.31 - 20.6, p ≤ 0.001) for mortality. Alcohol abuse (OR 2.15, 95% CI 1.19 - 3.88, p = 0.01) and cardiovascular diseases (OR 1.71, 95% CI 1.06 - 2.73, p = 0.03) were also significant risk predictors, while diabetes and psychiatric conditions remained non-significant. The model showed a substantial prognostic value of mortality (AUC = 0.70). Both HRQoL scores were compromised among the patients who died compared to those who lived throughout the follow-up period (for 15D score 0.75 vs. 0.80, p ≤ 0.001 and for AQ20 score 9.82 vs. 7.99, p ≤ 0.001).

## Discussion

The present patient cohort was hospital based representing all severity stages of the disease [26]. To analyse patients’ airway specific and generic HRQol we used two questionnaires. Both questionnaires are validated, well correlated and short enough to be integrated in clinical practise [25]. Our results show that cardiovascular diseases, diabetes, alcohol abuse, and different psychiatric diseases were more frequent in COPD patients than in controls. Like in previous reports, neither a particular co-morbidity nor the total number of co-morbidities correlated to the severity of airway obstruction [37]. However, co-morbidities were the most important determinants of HRQoL in subjects with mild and moderate severe obstruction. Only the presence of severe obstruction (FEV_1_ < 40%) was a significant contributor of HRQoL. Using the multivariate regression models, we found that co-morbidities were associated both with general and respiratory specific HRQoL, the strongest effects being observed for psychiatric conditions that needed regular medications and alcohol abuse. The co-morbidities were diagnosed typically years before the HRQoL-measure at study baseline, and thus could be considered as risk factors of poor HRQoL. Although this suggests causality, it cannot be confirmed in this cross-sectional study. The present study cohort reflects the heterogeneity found in COPD at large. The patients represented both genders and all severity stages of airway obstruction, and various patterns of co-morbidities were present, including cardiovascular diseases, chronic atrial fibrillation, hypertension, diabetes, obesity, psychiatric conditions, cancer, and alcohol abuse, confirming previous reports on multimorbidity in COPD [4,38].

Accumulation of comorbidities into the present COPD cohort was less than in some previous reports. In the present data set the diagnoses are based on retrospective medical records, but COPD patients may go under diagnosed when the symptoms of co-existing disease are not recognised or the symptoms are considered adverse events of COPD medication. Underestimation of co-existing diseases would thus weaken the role of co-morbidities explaining the variability found in HRQoL. Two to three times higher risk of cardiovascular diseases has been reported in several COPD populations [38,39]. Incompatibilities in the diagnostic criteria and differences in characteristics of COPD populations may explain to some extent the inconsistencies between studies. Preventive care for example in coronary disease has become more active over time which might explain why COPD patients were at significant risk of lung cancer, heart failure, and death, but not at risk of myocardial infarction [40]. In the present COPD cohort the diagnoses were given by a specialist and gathered from medical records. The diagnoses can be considered more plausible than self-reported often used in previous reports. The prevalence may also reflect recruitment bias, i.e. if the most seriously ill patients did not participate. Excessive alcohol consumption was pronounced among COPD patients. Compared to elderly Finns in general, alcohol abuse was 4.5 fold more frequent among women and 2.3 fold more frequent among men with COPD [41]. Excess alcohol use has been found also in other COPD populations [42]. Data on chronic atrial fibrillation and diffusing capacity were not available for all, but the univariate model suggested that chronic atrial fibrillation would harm HRQoL (crude OR 2.4, 95% CI 1.00-6.0, p ≤ 0.05), while impaired diffusing capacity did not. Prevalence of hypertension (41%) and chronic atrial fibrillation (6%) were at the same level as in general age and gender matched population in Finland [43].

Like in many previous studies, lung function alone was not a strong predictor of either respiratory or general HRQoL [22,44,45]. Literature on co-morbidities as determinants of HRQoL in COPD is rather sparse. Using St George’s Respiratory Questionnaire (SGRQ) in primary health care COPD population (N = 1817) it has been shown that patient with >3 co-morbidities had poorer HRQoL than patients with 1–2 comorbidities [46]. In patients with <3 co-morbidities the generic instrument was more sensitive than SGRQ suggesting that respiratory specific HRQoL is not influenced by the presence of low levels of co-morbidity. In another study (N = 326 COPD patients) the presence of dyspnoea and exacerbation, depression was the most important contributor to HRQoL impairment measured by SGRQ, whereas other comorbidities had only limited impact [47]. When 23 co-morbid diseases were analysed in a COPD cohort (N = 659, matching age and slightly more advanced disease than in the present study), the presence of three or more co-morbid diseases was more predictive of HRQoL than any demographic variable or physiological measurement [45]. Insomnia, however, was the only separate disease that correlated to general HRQoL. In another study by Putcha et al. (N = 843), four out of thirteen comorbid diseases (congestive heart failure, arthritis, diabetes, incontinence/prostate disease) associated independently with generic HRQoL [37]. All diagnostic information was based on self-reported data and the severity of COPD was not known in the latter study. These study designs included depression, but no other psychiatric or any addictive diseases like in our study. COPD patients in the present study displayed a large scale of psychiatric symptoms from features of psychosis to rather mild anxiety or depression. Not all of them had received a thorough psychiatric evaluation and thus the exact nature of the disease remained unknown, but the common nominator was the use of regular psychiatric medication. Psychiatric conditions (OR for low HRQoL for 15D 4.7 and for AQ20 2.0) and alcohol abuse (OR for 15D 2.3 and for AQ20 3.0) both had a strong impact on HRQoL that could be detected by generic and respiratory specific instruments. Our data also showed that diabetes affected generic HRQoL (OR 2.1) while cardiovascular diseases showed a similar trend even though not significant (OR 1.7). FEV_1_ was also a strong predictor of both generic and respiratory specific HRQoL, however the effect was significant only at late stages of disease (FEV_1_ < 40% of predicted).

Comparably with findings in the general population, cardiovascular morbidity was more prevalent among men than among women. Women reported significantly more respiratory symptoms even though their FEV_1_ was slightly better, as also reported earlier [48]. Regarding functional inabilities, women more often had sleeping difficulties, depression and distress while men suffered more often from impaired sexual performance.

At present, there is some evidence that HRQoL has predictive value for risk of exacerbations, use of health care resources, and mortality in COPD [49,50]. In the present study generic HRQoL was statistically (p ≤ 0.001) and clinically (MCID >0.03) poorer among patients who died within the next five years. If the patients’ 15D score was <0.815 his risk to die during the next five years was 2.3 times higher than among the rest of the patients (p ≤ 0.001). Correspondingly for AQ20 score >7.5 was associated with high mortality rate (OR = 2.16, p ≤ 0.001). The clinical variables, which predicted both poor HRQoL and mortality, were FEV_1_, alcohol abuse, and cardiovascular diseases. HRQoL has been considered a potential component to the new multidimensional health status –measures in COPD [12]. Our results also suggest that HRQoL might provide a broader insight in predicting mortality than FEV_1_ alone.

Recent studies have shown clearly that COPD can no longer be assessed as a disease only involving the lungs [51]. Even though COPD has pulmonary and extra-pulmonary manifestations both the generic and respiratory specific instruments gave surprisingly consistent results. The most important risk factors for poor HRQoL were identified by both instruments and by both analysis approaches applied. The risk factors for increased mortality were also partially overlapping and the HRQoL score was *per se* correlated with mortality. We have previously shown that when multiple HRQoL measures over five years are available for asthma and COPD patients constantly declining trends can be recognized with high probability [52]. In clinical practice the great benefit of the generic HRQoL instrument is that it allows the comparison of treatment interventions not only among COPD patients, but also in comparison to other patient groups suffering chronic diseases. Effectiveness of treatment options can be compared by using the ‘quality adjusted life years’ (QALY) measurement. Further this measurement allows us to consider the cost effectiveness of the treatment e.g. how much the treatments option costs per achieved QALY. It is an important additional feature in HRQoL studies, since disease burden of COPD is significant for public health care.

## Conclusion

Our results suggest that the effect of co-morbidities in COPD is underestimated in regard of HRQoL. Psychiatric conditions, alcohol abuse, cardiovascular disease and lung function are comparably important in predicting the HRQoL of a COPD patient. These findings are further underlined by the higher prevalence of these conditions in this hospital based COPD cohort, when compared to age and gender matched general population. We chose for the study two short and easy to fill-in questionnaires and thus applicable also in clinical practice. The instruments have been validated in COPD and they have been shown to correlate to other more complex questionnaires. Being generic, 15D better captured the impact of co-morbidities while respiratory specific AQ20 was more sensitive to impaired lung function. Five-year mortality was significantly higher in patients with poor HRQoL measure at study baseline.

## Competing interests

Jukka Koskela (JK) has no competing interests. Maritta Kilpeläinen (MK) has no competing interests. Henna Kupiainen (HK) has no competing interests. Witold Mazur (WM) has no competing interests. Harri Sintonen (HS) is the developer of the 15D and obtains royalties from electronic versions of the instrument. Marike Boezen (MB) has no competing interests. Ari Lindqvist (AL) has no competing interests. The University of Groningen has received money for Professor Postma (DP) regarding an unrestricted educational grant for research from Astra Zeneca, Chiesi. Travel to ERS and/or ATS has been partially funded by Astra Zeneca, Chiesi, GSK, Takeda. Fees for consultancies were given to the University of Groningen by Astra Zeneca, Boehringer Ingelheim, Chiesi, GSK, Takeda and REVA. Travel and lectures in China paid by Chiesi. Tarja Laitinen (TL) has no competing interests.

## Authors’ contributions

JK carried out statistical analysis of data and drafted the manuscript. MK participated in acquisition of data and interpretation of the results. HK participated in acquisition of data. WM helped in drafting the manuscript. HS participated in interpretation of the results and drafting of the manuscript. MB helped in statistical analysis of the data. AL participated in acquisition of data. DP helped in interpretation of the results and drafting the manuscript. TL designed the study approach, analysis and interpretation of data. All authors read and approved the final manuscript.

## Pre-publication history

The pre-publication history for this paper can be accessed here:

http://www.biomedcentral.com/1471-2466/14/102/prepub

## Supplementary Material

Additional file 1: Table S1 Dimensions of HRQoL determined by 15D that differed significantly between males and females. **Table S2** Dimensions of HRQoL determined by AQ20 that differed significantly between males and females. **Table S3** Risk factors for mortality among COPD patients. Click here for file

## References

[B1] Agusti A Soriano JB COPD as a systemic disease COPD 2008 5 2 133 138 doi: 10.1080/15412550801941349; 10.1080/15412550801941349 10.1080/15412550801941349 18415812

[B2] Fabbri LM Luppi F Beghe B Rabe KF Complex chronic comorbidities of COPD Eur Respir J 2008 31 1 204 212 doi: 10.1183/09031936.00114307; 10.1183/09031936.00114307 10.1183/09031936.00114307 18166598

[B3] van Manen JG Bindels PJ IJzermans CJ van der Zee JS Bottema BJ Schade E Prevalence of comorbidity in patients with a chronic airway obstruction and controls over the age of 40 J Clin Epidemiol 2001 54 3 287 293 10.1016/S0895-4356(01)00346-8 11223326

[B4] MacNee W Systemic inflammatory biomarkers and co-morbidities of chronic obstructive pulmonary disease Ann Med 2013 45 3 291 300 doi: 10.3109/07853890.2012.732703; 10.3109/07853890.2012.732703 10.3109/07853890.2012.732703 23110517

[B5] Sin DD Anthonisen NR Soriano JB Agusti AG Mortality in COPD: role of comorbidities Eur Respir J 2006 28 6 1245 1257 doi: 10.1183/09031936.00133805 10.1183/09031936.00133805 17138679

[B6] Mannino DM Thorn D Swensen A Holguin F Prevalence and outcomes of diabetes, hypertension and cardiovascular disease in COPD Eur Respir J 2008 32 4 962 969 doi: 10.1183/09031936.00012408; 10.1183/09031936.00012408 10.1183/09031936.00012408 18579551

[B7] Vollmer WM Gislason T Burney P Enright PL Gulsvik A Kocabas A Buist AS Comparison of spirometry criteria for the diagnosis of COPD: results from the BOLD study Eur Respir J 2009 34 3 588 597 doi: 10.1183/09031936.00164608; 10.1183/09031936.00164608 10.1183/09031936.00164608 19460786 PMC3334278

[B8] Ford ES Wheaton AG Mannino DM Presley-Cantrell L Li C Croft JB Elevated cardiovascular risk among adults with obstructive and restrictive airway functioning in the United States: a cross-sectional study of the national health and nutrition examination survey from 2007–2010 Respir Res 2012 13 115-9921-13-115 doi: 10.1186/1465-9921-13-115; 10.1186/1465-9921-13-115 23237325 10.1186/1465-9921-13-115 PMC3546884

[B9] Shavelle RM Paculdo DR Kush SJ Mannino DM Strauss DJ Life expectancy and years of life lost in chronic obstructive pulmonary disease: findings from the NHANES III follow-up study Int J Chron Obstruct Pulmon Dis 2009 4 137 148 19436692 10.2147/copd.s5237 PMC2672796

[B10] Mannino DM Buist AS Petty TL Enright PL Redd SC Lung function and mortality in the United States: data from the first national health and nutrition examination survey follow up study Thorax 2003 58 5 388 393 10.1136/thorax.58.5.388 12728157 PMC1746680

[B11] Alfageme I Reyes N Merino M Reina A Gallego J Lima J Palacios Z The effect of airflow limitation on the cause of death in patients with COPD Chron Respir Dis 2010 7 3 135 145 doi: 10.1177/1479972310368692; 10.1177/1479972310368692 10.1177/1479972310368692 20688891

[B12] Domingo-Salvany A Lamarca R Ferrer M Garcia-Aymerich J Alonso J Félez M Khalaf A Marrades RM Monsó E Serra-Batlles J Antó JM Health-related quality of life and mortality in male patients with chronic obstructive pulmonary disease Am J Respir Crit Care Med 2002 166 5 680 685 doi: 10.1164/rccm.2112043 10.1164/rccm.2112043 12204865

[B13] Schols AM Slangen J Volovics L Wouters EF Weight loss is a reversible factor in the prognosis of chronic obstructive pulmonary disease Am J Respir Crit Care Med 1998 157 6 Pt 1 1791 1797 9620907 10.1164/ajrccm.157.6.9705017

[B14] Konecny T Somers K Orban M Koshino Y Lennon RJ Scanlon PD Rihal CS Interactions between COPD and outcomes after percutaneous coronary intervention Chest 2010 138 3 621 627 doi: 10.1378/chest.10-0300; 10.1378/chest.10-0300 10.1378/chest.10-0300 20382719

[B15] Sin DD Man SF Chronic obstructive pulmonary disease as a risk factor for cardiovascular morbidity and mortality Proc Am Thorac Soc 2005 2 1 8 11 doi: 10.1513/pats.200404-032MS 10.1513/pats.200404-032MS 16113462

[B16] Vestbo J Anderson W Coxson HO Crim C Dawber F Edwards L Hagan G Knobil K Lomas DA MacNee W Silverman EK Tal-Singer R ECLIPSE investigators: Evaluation of COPD Longitudinally to Identify Predictive Surrogate End-points (ECLIPSE) Eur Respir J 2008 31 4 869 873 10.1183/09031936.00111707 18216052

[B17] Calverley PM Anderson JA Celli B Ferguson GT Jenkins C Jones PW Yates JC Vestbo J TORCH investigators Salmeterol and fluticasone propionate and survival in chronic obstructive pulmonary disease N Engl J Med 2007 356 8 775 789 doi: 10.1056/NEJMoa063070 10.1056/NEJMoa063070 17314337

[B18] Decramer M Celli B Kesten S Lystig T Mehra S Tashkin DP UPLIFT investigators Effect of tiotropium on outcomes in patients with moderate chronic obstructive pulmonary disease (UPLIFT): a prespecified subgroup analysis of a randomised controlled trial Lancet 2009 374 9696 1171 1178 doi: 10.1016/S0140-6736(09)61298-8; 10.1016/S0140-6736(09)61298-8 10.1016/S0140-6736(09)61298-8 19716598

[B19] de Torres JP Casanova C Hernandez C Abreu J Aguirre-Jaime A Celli BR Gender and COPD in patients attending a pulmonary clinic Chest 2005 128 4 2012 2016 doi: 10.1378/chest.128.4.2012 10.1378/chest.128.4.2012 16236849

[B20] Almagro P Lopez Garcia F Cabrera F Montero L Morchón D Díez J Soriano J Grupo Epoc De La Sociedad Española De Medicina Interna Comorbidity and gender-related differences in patients hospitalized for COPD. the ECCO study Respir Med 2010 104 2 253 259 doi: 10.1016/j.rmed.2009.09.019 10.1016/j.rmed.2009.09.019 19879744

[B21] Nurmatov U Buckingham S Kendall M Murray SA White P Sheikh A Pinnock H Effectiveness of holistic interventions for people with severe chronic obstructive pulmonary disease: systematic review of controlled clinical trials PLoS One 2012 7 10 e46433 doi: 10.1371/journal.pone.0046433; 10.1371/journal.pone.0046433 10.1371/journal.pone.0046433 23110052 PMC3479091

[B22] Hajiro T Nishimura K Jones PW Tsukino M Ikeda A Koyama H Izumi T A novel, short, and simple questionnaire to measure health-related quality of life in patients with chronic obstructive pulmonary disease Am J Respir Crit Care Med 1999 159 6 1874 1878 doi: 10.1164/ajrccm.159.6.9807097 10.1164/ajrccm.159.6.9807097 10351933

[B23] Saarni SI Harkanen T Sintonen H Suvisaari J Koskinen S Aromaa A Lönnqvist J The impact of 29 chronic conditions on health-related quality of life: a general population survey in finland using 15D and EQ-5D Qual Life Res 2006 15 8 1403 1414 doi: 10.1007/s11136-006-0020-1 10.1007/s11136-006-0020-1 16960751

[B24] Jones PW A self-complete measure of health status for chronic airflow limitation. the St. George’s respiratory questionnaire Am Rev Respir Dis 1992 145 6 1321 1327 10.1164/ajrccm/145.6.1321 1595997

[B25] Mazur W Kupiainen H Pitkaniemi J Kilpeläinen M Sintonen H Lindqvist A Kinnula VL Laitinen T Comparison between the disease-specific airways questionnaire 20 and the generic 15D instruments in COPD Health Qual Life Outcome 2011 9 4 doi: 10.1186/1477-7525-9-4 10.1186/1477-7525-9-4 PMC3031191 21235818

[B26] Laitinen T Hodgson U Kupiainen H Tammilehto L Haahtela T Kilpeläinen M Lindqvist A Kinnula VL Real-world clinical data identifies gender-related profiles in chronic obstructive pulmonary disease COPD 2009 6 4 256 262 10.1080/15412550903051799 19811384

[B27] Barley EA Quirk FH Jones PW Asthma health status measurement in clinical practice: validity of a new short and simple instrument Respir Med 1998 92 10 1207 1214 10.1016/S0954-6111(98)90423-1 9926151

[B28] Alemayehu B Aubert RE Feifer RA Paul LD Comparative analysis of two quality-of-life instruments for patients with chronic obstructive pulmonary disease Value Health 2002 5 5 437 442 10.1046/J.1524-4733.2002.55151.x 12201861

[B29] Sintonen H The 15D instrument of health-related quality of life: properties and applications Ann Med 2001 33 5 328 336 10.3109/07853890109002086 11491191

[B30] Ritva K Pekka R Harri S Agreement between a generic and disease-specific quality-of-life instrument: the 15D and the SGRQ in asthmatic patients Qual Life Res 2000 9 9 997 1003 10.1023/A:1016698818258 11332228

[B31] Stavem K Reliability, validity and responsiveness of two multiattribute utility measures in patients with chronic obstructive pulmonary disease Qual Life Res 1999 8 1–2 45 54 10457737 10.1023/a:1026475531996

[B32] Viljanen AA Halttunen PK Kreus KE Viljanen BC Spirometric studies in non-smoking, healthy adults Scand J Clin Lab Invest Suppl 1982 159 5 20 6957974

[B33] Heistaro S Methodology Report, Health 2000 Survey: 2008 2008 Helsinki: National Public Health Institute

[B34] Bestall JC Paul EA Garrod R Garnham R Jones PW Wedzicha JA Usefulness of the Medical Research Council (MRC) dyspnoea scale as a measure of disability in patients with chronic obstructive pulmonary disease Thorax 54 581 586 doi: 10.1136/thx.54.7.581 10377201 10.1136/thx.54.7.581 PMC1745516

[B35] Sintonen H Outcome measurement in acid-related diseases Pharmacoeconomics 1994 5 3 17 26 http://dx.doi.org/10.2165/00019053-199400053-00005. doi: 10.2165/00019053-199400053-00005

[B36] Vestbo J Hurd SS Agusti AG Jones PW Vogelmeier C Anzueto A Barnes PJ Fabbri LM Martinez FJ Nishimura M Stockley RA Sin DD Rodriguez-Roisin R Global strategy for the diagnosis, management, and prevention of chronic obstructive pulmonary disease: GOLD executive summary Am J Respir Crit Care Med 2013 187 4 347 365 10.1164/rccm.201204-0596PP 22878278

[B37] Putcha N Puhan MA Hansel NN Drummond MB Boyd CM Impact of co-morbidities on self-rated health in self-reported COPD: an analysis of NHANES 2001–2008 COPD 2013 10 3 324 332 doi: 10.3109/15412555.2012.744963; 10.3109/15412555.2012.744963 10.3109/15412555.2012.744963 23713595 PMC4459792

[B38] Feary JR Rodrigues LC Smith CJ Hubbard RB Gibson JE Prevalence of major comorbidities in subjects with COPD and incidence of myocardial infarction and stroke: a comprehensive analysis using data from primary care Thorax 2010 65 11 956 962 doi: 10.1136/thx.2009.128082; 10.1136/thx.2009.128082 10.1136/thx.2009.128082 20871122

[B39] Lusuardi M Garuti G Massobrio M Spagnolatti L Bendinelli S Heart and lungs in COPD. close friends in real life–separate in daily medical practice? Monaldi Arch Chest Dis 2008 69 1 11 17 18507194 10.4081/monaldi.2008.406

[B40] Rodriguez LA Wallander MA Martin-Merino E Johansson S Heart failure, myocardial infarction, lung cancer and death in COPD patients: A UK primary care study Respir Med 2010 104 11 1691 1699 doi: 10.1016/j.rmed.2010.04.018; 10.1016/j.rmed.2010.04.018 10.1016/j.rmed.2010.04.018 20483577

[B41] Halme JT Seppa K Alho H Pirkola S Poikolainen K Lönnqvist J Aalto M Hazardous drinking: prevalence and associations in the finnish general population Alcohol Clin Exp Res 2008 32 9 1615 1622 doi: 10.1111/j.1530-0277.2008.00740.x; 10.1111/j.1530-0277.2008.00740.x 10.1111/j.1530-0277.2008.00740.x 18616689

[B42] Ryan M Merrick EL Hodgkin D Horgan CM Garnick DW Panas L Ritter G Blow FC Saitz R Drinking patterns of older adults with chronic medical conditions J Gen Intern Med 2013 doi: 10.1007/s11606-013-2409-1 10.1007/s11606-013-2409-1 PMC3785666 23609178

[B43] Kastarinen M Antikainen R Peltonen M Laatikainen T Barengo NC Jula A Salomaa V Jousilahti P Nissinen A Vartiainen E Tuomilehto J Prevalence, awareness and treatment of hypertension in Finland during 1982–2007 J Hypertens 2009 27 8 1552 1559 doi: 10.1097/HJH.0b013e32832c41cd; 10.1097/HJH.0b013e32832c41cd 10.1097/HJH.0b013e32832c41cd 19412128

[B44] Ferrer M Alonso J Morera J Marrades RM Khalaf A Aguar MC Plaza V Prieto L Antó JM Chronic obstructive pulmonary disease stage and health-related quality of life. the quality of life of chronic obstructive pulmonary disease study group Ann Intern Med 1997 127 12 1072 1079 10.7326/0003-4819-127-12-199712150-00003 9412309

[B45] van Manen JG Bindels PJ Dekker EW Ijzermans CJ Bottema BJ van der Zee JS Schadé E Added value of co-morbidity in predicting health-related quality of life in COPD patients Respir Med 2001 95 6 496 504 10.1053/rmed.2001.1077 11421508

[B46] Jones PW Brusselle G Dal Negro RW Ferrer M Kardos P Levy ML Perez T Soler-Cataluña JJ van der Molen T Adamek L Banik N Health-related quality of life in patients by COPD severity within primary care in Europe Respir Med 2011 105 1 57 66 doi: 10.1016/j.rmed.2010.09.004 10.1016/j.rmed.2010.09.004 20932736

[B47] Burgel PR Escamilla R Perez T Carré P Caillaud D Chanez P Pinet C Jebrak G Brinchault G Court-Fortune I Paillasseur JL Roche N INITIATIVES BPCO Scientific Committee Impact of comorbidities on COPD-specific health-related quality of life Respir Med 2013 107 2 233 241 doi: 10.1016/j.rmed.2012.10.002 10.1016/j.rmed.2012.10.002 23098687

[B48] Watson L Schouten JP Löfdahl CG Pride NB Laitinen LA Postma DS Predictors of COPD symptoms: does the sex of the patient matter? Eur Respir J 2006 28 2 311 318 10.1183/09031936.06.00055805 16707516

[B49] Blanco-Aparicio M Vazquez I Pita-Fernandez S Pertega-Diaz S Verea-Hernando H Utility of brief questionnaires of health-related quality of life (airways questionnaire 20 and clinical COPD questionnaire) to predict exacerbations in patients with asthma and COPD Health Qual Life Outcome 2013 11 85-7525-11-85 doi: 10.1186/1477-7525-11-85; 10.1186/1477-7525-11-85 10.1186/1477-7525-11-85 PMC3701555 23706146

[B50] Ryynanen OP Soini EJ Lindqvist A Kilpelainen M Laitinen T Bayesian predictors of very poor health related quality of life and mortality in patients with COPD BMC Med Inform Decis Mak 2013 13 34-6947-13-34 doi: 10.1186/1472-6947-13-34; 10.1186/1472-6947-13-34 23496851 10.1186/1472-6947-13-34 PMC3610236

[B51] Cazzola M MacNee W Martinez FJ Rabe KF Franciosi LG Barnes PJ Brusasco V Burge PS Calverley PM Celli BR Jones PW Mahler DA Make B Miravitlles M Page CP Palange P Parr D Pistolesi M Rennard SI Rutten-van Mölken MP Stockley R Sullivan SD Wedzicha JA Wouters EF American Thoracic Society; European Respiratory Society Task Force on outcomes of COPD Outcomes for COPD pharmacological trials: from lung function to biomarkers Eur Respir J 2008 31 2 416 469 doi: 10.1183/09031936.00099306; 10.1183/09031936.00099306 10.1183/09031936.00099306 18238951

[B52] Koskela J Kupiainen H Kilpelainen M Lindqvist A Sintonen H Pitkäniemi J Laitinen T Longitudinal HRQoL shows divergent trends and identifies constant decliners in asthma and COPD Respir Med 2014 108 463 471 10.1016/j.rmed.2013.12.001 24388549

