# Supplementary material for: Co-morbidities are the key nominators of the health related quality of life in mild and moderate COPD
Source: BMC Pulm Med. 2014 Jun 19;14:102. doi: 10.1186/1471-2466-14-102 (PMC4229911; doi:10.1186/1471-2466-14-102)
Supplement: Additional file 1: Table S1 — Dimensions of HRQoL determined by 15D that differed significantly between males and females. Table S2 Dimensions of HRQoL determined by AQ20 that differed significantly between males and females. Table S3 Risk factors for mortality among COPD patients. [file 1471-2466-14-102-S1.docx]

**Supplementantary information**

**Table S1.** Dimensions of HRQoL determined by 15D that differed significantly between males and females.

| **Dimension of HRQoL** | **Score**  **Mean (SD)** | | **P value^1^** |
| --- | --- | --- | --- |
|  | **Men** | **Women** |  |
| Hearing | 0.90 (0.15) | 0.93 (0.14) | 0.002 |
| Sleep | 0.75 (0.22) | 0.71 (0.24) | 0.03 |
| Depression | 0.85 (0.18) | 0.79 (0.18) | <0.0001 |
| Distress | 0.83 (0.18) | 0.79 (0.20) | 0.004 |
| Sex | 0.62 (0.29) | 0.76 (0.28) | <0.0001 |

^1^ Mann-Whitney U test

**Table S2.** Dimensions of HRQoL determined by AQ20 that differed significantly between males and females.

| **Dimension of HRQoL** | **Score**  **Mean (SD)** | | **P value^1^** |
| --- | --- | --- | --- |
|  | **Men** | **Women** |  |
| AQ2: Because of your chest trouble do you often feel restless? | 0.39 (0.49) | 0.48 (0.50) | p=0.02 |
| AQ4: Do you worry when going to a friend’s house that there might be something there that will set off an attack of chest trouble? | 0.13 (0.34) | 0.18 (0.39) | p=0.05 |
| AQ5: Do you suffer from chest symptoms as a result of exposure to strong smells, cigarette smoke or perfume? | 0.45 (0.50) | 0.56 (0.50) | p<0.01 |
| AQ6: Is your partner bothered by your chest trouble? | 0.34 (0.47) | 0.27 (0.44) | p=0.05 |
| AQ8: Do you worry about the long term effects on your health of the drugs that you have to take because of your chest trouble? | 0.28 (0.45) | 0.38 (0.49) | p<0.01 |
| AQ9: Does getting emotionally upset make your chest trouble worse? | 0.45 (0.50) | 0.54 (0.50) | p=0.01 |
| AQ13: Because of your chest trouble do you suffer from breathlessness doing housework? | 0.42 (0.49) | 0.56 (0.50) | p<0.001 |
| AQ15: Because of your chest trouble do you suffer from breathlessness when you laugh? | 0.08 (0.28) | 0.21, (0.41) | p<0.01 |
| AQ18: Do you feel drained after a cold because of your chest trouble? | 0.50 (0.50) | 0.63, (0.48) | p<0.01 |
| AQ19: Do you have a feeling of chest heaviness? | 0.44 (0.50) | 0.57 (0.50) | p<0.01 |

^1^ Mann-Whitney U test

Table S3. Risk factors for mortality among COPD patients.

| **Patient**  **Characteristics** | **Adjusted**  **OR** | **95% CI** | **P-value** |
| --- | --- | --- | --- |
| FEV_1_ baseline % of predicted  >80  65-80  40-64  <40 | 1.00  2.01  2.58  **6.90** | 0.64-6.33  0.88-7.63  **2.31-20.6** | 0.23  0.09  **<0.001** |
| Diabetes  No  Yes | 1.00  1.60 | 0.89-2.88 | 0.11 |
| Cardiovascular Disease^1^  No  Yes | 1.00  **1.71** | **1.06-2.74** | **0.03** |
| Psychiatric conditions  No  Yes | 1.00  1.09 | 0.67-1.79 | 0.72 |
| Alcohol abuse  No  Yes | 1.00  **2.15** | **1.20-3.88** | **0.01** |
